# Herpetic Geometric Glossitis in an Immunocompetent Patient: An Atypical Presentation of Oral Herpes Simplex Virus (HSV) Infection

**DOI:** 10.7759/cureus.111663

**Published:** 2026-06-28

**Authors:** Steven T Rivera, Laura A Chachula, Paul S Hahn

**Affiliations:** 1 Dermatology, San Antonio Uniformed Services Health Education Consortium, San Antonio, USA

**Keywords:** atypical herpetic infection, herpes simplex, herpes simplex virus type 1, herpetic glossitis, hsv-1

## Abstract

Herpetic geometric glossitis (HGG) is a rare, atypical manifestation of herpes simplex virus type 1 (HSV-1) infection, most commonly reported in immunocompromised individuals. We report a case of a 43-year-old immunocompetent male presenting with edematous heme-crusted lips, fissured tongue, and severe oral pain, leading to significant oral intake limitation and subsequent inpatient admission. Dermatology was consulted for possible Stevens-Johnson syndrome, which was ruled out following evaluation.

Clinical findings were consistent with HGG, an uncommon presentation of HSV infection. The patient started empiric antiviral therapy, leading to symptom improvement, and HSV-1 polymerase chain reaction testing returned positive, confirming the diagnosis. This case highlights a rare presentation of HSV-1 in an immunocompetent host and emphasizes the importance of recognizing atypical morphologic variants to guide timely diagnosis and management.

## Introduction

Orofacial mucositis encompasses a broad spectrum of conditions involving inflammation of the oral mucosa. Common causes include herpes simplex virus (HSV), candidiasis, erythema multiforme (EM), and Mycoplasma-induced rash and mucositis (MIRM) [1-3]. HSV-1 is a frequent cause of recurrent oral mucosal disease, most commonly presenting as recurrent herpes labialis; however, clinical presentations can vary significantly. Herpetic geometric glossitis (HGG) is a rare, atypical manifestation of HSV infection characterized by fissured, linear, or geometric patterns involving the tongue, more often seen in the setting of immune compromise [4]. HGG was first described by Grossman et al. in 1993 in a patient with HIV [5]. Since then, only a handful of cases in immunocompetent patients have been reported; the rest include HIV-positive patients, patients being treated with systemic corticosteroids, a cardiac transplant patient, and a boy with acute myelogenous leukemia [4,6].

EM is another mucocutaneous condition frequently associated with HSV. Herpes-associated EM presents with numerous target lesions on acral extremities within 10 days of an oral or genital herpetic reactivation [7]. Patients with EM can present with significant hemorrhagic crusting of the lips mimicking severe cutaneous drug reactions such as Stevens-Johnson syndrome.

We report a rare case of HGG in an immunocompetent patient presenting with prominent lip involvement, raising concern for EM, highlighting diagnostic challenges and the importance of recognizing atypical HSV morphologies.

## Case presentation

**Figure 1:**
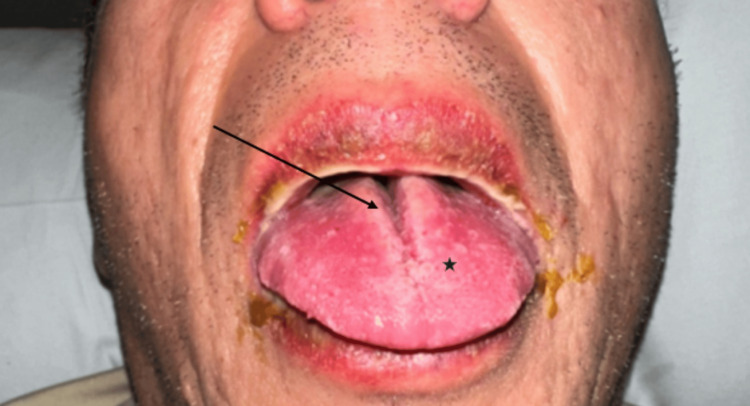
Initial exam on day 1 of admission. Linear longitudinal fissure with elevated borders on the central tongue (arrow). Multiple punched-out erythematous erosions on the anterior tongue (star). Hemorrhagic and honey colored crust on the upper and lower lips.

A 43-year-old male with a past medical history of anxiety, depression, and post-traumatic stress disorder presented with severe oral pain and swelling, limiting oral intake. The patient reported three episodes over the preceding six to eight months, with earlier episodes consisting of transient oral burning that resolved spontaneously. The most recent episode began acutely, awakening him from sleep with burning pain of the lips and tongue, followed by progressive swelling and fissuring of the tongue, and crusting of the lips (Figure 1). Pain worsened over several days, resulting in inability to tolerate solid food.

He denied fever, systemic symptoms, medication changes, new exposures, and recent upper respiratory infection. He had no history of immunosuppression. Physical examination revealed marked edema of the upper and lower lips with yellow crusting at the commissures. The tongue was enlarged with a prominent midline fissure and scalloped borders, along with areas of scrapable white plaques and surrounding erythema. No involvement of the buccal mucosa, gingiva, or palate was noted. There were no cutaneous, ocular, or genital lesions.

Laboratory evaluation, including complete blood count, metabolic panel, and inflammatory markers, was within normal limits. The patient was afebrile. Initial outpatient treatment with nystatin for presumed oral candidiasis did not result in improvement.

**Figure 2:**
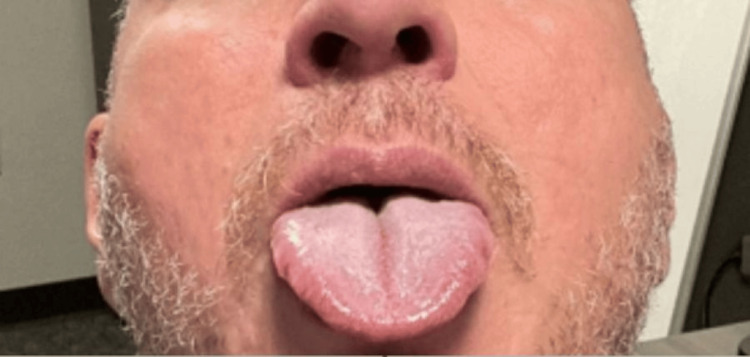
Resolution of swelling and fissuring of the tongue and crusting of the lips following treatment. Two weeks after completing valacyclovir.

Dermatology was consulted to rule out Stevens-Johnson syndrome and toxic epidermal necrolysis, although there was low suspicion given that the patient was stable, lacked cutaneous involvement, and was not on inciting medications. HSV-1/HSV-2 PCR testing was obtained from an oral swab, and empiric treatment with valacyclovir 1 g twice daily for seven days was initiated. The HSV-1 PCR returned positive, and the patient saw rapid improvement within days of starting valacyclovir and complete resolution two weeks after completing treatment (Figure 2).

## Discussion

This case represents a rare presentation of HSV-1 infection as HGG in an immunocompetent individual. HGG is characterized by fissured, linear, or geometric patterns of tongue involvement and has primarily been described in immunocompromised patients [4]. The presence of a deep midline fissure, a scalloped tongue, and irregular mucosal patterns in this patient is consistent with previously described morphologic features of this entity. In contrast to similar-appearing tongue lesions, HGG is associated with severe pain, tenderness, or burning sensation, as seen in our patient [8].

Intraoral HSV infections in adults frequently lack classic vesicular lesions and instead manifest as diffuse mucositis, erythema, swelling, or fissuring [7]. Additionally, HSV reactivation is associated with immune-mediated conditions such as EM [9]. In this case, the absence of cutaneous lesions and systemic symptoms made EM less likely [2,3]. MIRM was also considered in this patient, however the lack of recent respiratory illness and classic rash made this less favored.

This case underscores the importance of recognizing atypical HSV presentations, particularly HGG, even in immunocompetent individuals. Early diagnostic testing with HSV PCR is critical in establishing the diagnosis and guiding therapy; however, empiric antiviral treatment is appropriate when suspicion is high, given its favorable safety profile and potential to reduce morbidity. Systemic antiviral therapy follows the same treatment principles as with other oral HSV infections, with oral antivirals often used for immunocompetent individuals, such as in the case presented, and IV acyclovir used for more severe mucocutaneous involvement, often in those who are immunocompromised.

## Conclusions

HGG is a rare manifestation of HSV-1 infection that may occur even in immunocompetent individuals. This case highlights the importance of considering HSV in patients with recurrent oral mucositis and atypical tongue morphology. Recognition of this entity and early diagnostic testing can facilitate timely treatment and improve patient outcomes.
